# Ethical Oversight and Social Licensing of Portable MRI Research

**DOI:** 10.1017/jme.2024.166

**Published:** 2024

**Authors:** Barbara J. Evans

**Affiliations:** 1: UNIVERSITY OF FLORIDA, GAINESVILLE, FL, USA

**Keywords:** Portable MRI, Neurological Research, HIPAA Privacy Rule, Common Rule, FDA Investigational Device Exemption/IDE, Social Licensing/Social License/Social Licence

## Abstract

This article explores two questions: (1) whether portable MRI research might escape regulatory oversight altogether under existing U.S. privacy and research ethical frameworks, leaving research participants without adequate protections, and (2) whether existing regulatory frameworks, when they do apply, can guard society’s broader interest in ensuring that portable MRI research pursues socially beneficial, ethically sound aims that minimize the potential for externalities affecting nonparticipating individuals and groups, who might be stigmatized or otherwise harmed even if they decline participation in the research.

## Introduction

Portable, low-field MRI opens the possibility of conducting brain research in field settings such as homes, schools, retail stores, courtrooms, sports arenas, and other locations outside academic medical centers. Bringing brain research to the people, instead of requiring research participants to travel to fixed MRI scanners at academic hospitals, could diversify databases and reduce bias in neuroscience.^1^ Portable MRI could let nonmedical researchers (including citizen scientists) explore how the ultimate black box — the brain — affects human performance in real-world situations that are difficult to replicate in traditional, hospital-bound research environments.

Potential research uses, many of which are still speculative, include educational research and studies of juror decision-making, economic decisions and consumer choice, voting behaviors, and the creation of art.^2^ Scholars suggest that emerging fields of “neurolaw, neuroeconomics, educational neuroscience, neuropolitics, neuromarketing, neurophilosophy, and neurosociology may increasingly integrate scanning into their research.”^3^ At the same time, scholars caution that current ethical and regulatory frameworks have gaps that might lead to incomplete or ineffective protection of future participants in pMRI research.^4^

This article offers a cautious and somewhat skeptical counterpoint to the view that pMRI will rapidly infiltrate nonmedical academic disciplines and open a new era of neuro-*everything* research. A low-field pMRI scanner, estimated in 2023 to have an upfront cost around $250,000,^5^ costs far less than some of its traditional counterparts but is still not something most professors would be able to purchase for their offices. Tinkerers can build their own open-source scanners using open-source software and available hardware parts, as in the recent Uganda demonstration Shen et al. describe elsewhere in this issue.^6^ However, “FDA [the U.S. Food and Drug Administration] may consider those who build open-source scanners to be ‘manufacturers’ subject to FDA regulations.”^7^ FDA almost surely *will* do so if the tinkerers are located in, or exporting to, the United States and intend to use their homemade devices to experiment on anybody other than themselves. Similar issues arise with biohacking and consumer genetic technologies and shed light on how hard it may be to regulate citizen science with open-source portable MRI.^8^ Studies of consumer genomics suggest that FDA has considerable legal authority to regulate do-it-yourself scientists but would face practical challenges in regulating thousands of small manufacturers and researchers.^9^ Agencies like FDA were designed in the 20th century to regulate “a small number of large companies” and research institutions.^10^ Technologies like pMRI undermine the centralized industry structures that yesterday’s regulations presume.^11^

Imagine a future in which market researchers can prove (with sound scientific evidence!) that their brand of peanut butter lights up the pleasure centers in our brains better than the competing brand, so that consumers need never again endure the hedonic injury of choosing the wrong brand of peanut butter. Would such “science” benefit the public, and who should be the judge of that?

Despite the regulatory gaps — which are very real — this article argues that a variety of cost and regulatory barriers will constrain the pace at which educators, philosophers, lawyers, marketers, and other medical laypeople incorporate pMRI machines into their day-to-day professional activities and research. Current uses of pMRI cited in a recent survey reflect routine clinical uses of brain scanning and traditional medical research projects, novel only in that they now can be performed in non-traditional settings.^12^ They do not suggest wide use of pMRI in nonmedical research projects at this time.^13^ Yet we all have seen, in our recent lifetimes, how quickly the cost and availability of new technologies can change. Even skeptics accept that pMRI may, at some point, be used for a variety of nonmedical research projects, which are the main focus of this article.

Some nonmedical research uses of pMRI might be ethical, scientifically sound, and socially beneficial, yet others will raise thornier ethical concerns. Portable scanners already make it possible to estimate brain volumes “in children 6 weeks to 16 years of age … in almost any setting.”^14^ Deploying these systems in schools might help researchers identify educational and social interventions that effectively boost brain health and academic performance in struggling students. Yet it also might allow educators to stigmatize young children and consign them to an invidious “small-brained” classification in which their futures are simply written off. It is worth remembering that Ivan Turgenev’s brain, at autopsy, weighed 2.021 kilograms, whereas Anatole France’s brain was a mere half that (1.017 kilogram), yet both authors delighted many readers.^15^ How many Anatole Frances might educators write off if pMRI scanners are placed in future classrooms?

Future nonmedical brain research, even when not ethically troubling, might sometimes lack scientific merit or be targeted at problems with low value to society. Imagine a future in which market researchers can prove (with sound scientific evidence!) that their brand of peanut butter lights up the pleasure centers in our brains better than the competing brand, so that consumers need never again endure the hedonic injury of choosing the wrong brand of peanut butter. Would such “science” benefit the public, and who should be the judge of that?

This article explores whether current U.S. research regulatory structures are tailored to the challenges of using pMRI in field settings for nonmedical research purposes such as sociological, educational, or marketing studies. There are two questions: (1) whether this research might escape regulatory oversight altogether under existing U.S. privacy and research ethical frameworks, leaving research participants without adequate protections, and (2) whether the existing frameworks, when they do apply, offer meaningful protection against ethically questionable, scientifically dubious uses of portable MRI technology that threaten broader harms to society at large.

## I. Can Current Regulations Protect Portable MRI Research Participants?

Three major U.S. federal research regulatory frameworks potentially protect human participants in portable MRI research: the Federal Policy for Protection of Human Subjects (Common Rule);^16^ the Health Insurance Portability and Accountability Act^17^ (HIPAA) Privacy Rule,^18^ a medical privacy regulation that applies to some but not all biomedical research activities; and FDA’s research oversight framework, which potentially comes into play because portable MRI scanners are subject to FDA regulation as medical devices.^19^ This part explores whether portable MRI research could fall into jurisdictional gaps in these regulations, leaving research participants with insufficient ethical and privacy protections. To summarize, these concerns are valid, especially for the Common Rule and HIPAA Privacy Rule. Fortunately, FDA has at least some jurisdiction to regulate research uses of portable MRI, even in basic scientific studies pursuing nonmedical research aims. However, this analysis confirms that there is a real risk that some research participants may fall into regulatory gaps and be left unprotected.

### A. Risk Protections Under the Common Rule and HIPAA Privacy Rule

Field-based portable MRI research carries risks that warrant dependable privacy and ethical protections. These risks include privacy risks and potential risks of physical injuries.

As for the privacy risks, neuroimages are potentially re-identifiable even when researchers make efforts to anonymize them,^20^ and these risks are only expected to intensify as artificial intelligence (AI) algorithms grow more adept at reidentifying image data. Another privacy concern is that moving research outside the traditional biomedical research context exposes participants to a host of new data handlers — medical device manufacturers, information processors, data storage and transmission providers, and nonmedical research personnel — who may not be bound by the default privacy and confidentiality norms governing physicians, nurses, and trainees working within licensed health care institutions.^21^ Portable MRI research thus may deviate from research participants’ traditional expectations of research privacy.

As for safety risks, portable MRI scanners, although safer than many other neuroimaging technologies, are not altogether risk-free, as Shen et al. discuss elsewhere in this issue.^22^ Unlike genetic and other *in vitro* diagnostics that only require a low-risk blood draw or buccal swab to collect specimens, MRIs are *in vivo* diagnostics that study parts of the body *in situ* — within a living human being — by exposing research participants to energy flows.^23^ To be sure, portable MRI scanners employ much lower magnetic fields (fractions of 1 Tesla) than standard and high-field devices operating at 1.5 Tesla and above.^24^ Low-field portable MRI scanners reduce the risks seen with higher magnetic fields, such as forces on metallic implants, tissue heating, vertigo, nausea and other risks Hoff et al. observed in 7-Tesla devices used in clinical care.^25^

While the lower field strength is comforting, two caveats are warranted. First, the reduction in magnetic field exposure comes at the cost of lower image quality, which can subject research participants to other safety risks if, for example, possibly-less-accurate scans are returned to them or used to make decisions about the interventions they will receive during the research. Second — and FDA has stressed this point again and again — research can pose significant risk to research participants, even when it uses devices that, in themselves, are relatively safe. In research oversight, “[t]he risk determination is based on the proposed use of a device in an investigation, and not on the device alone.”^26^ Even when FDA has determined that a device is NSR, or “nonsignificant risk,” that does not imply that research using the device also poses non-significant safety risks to participants, “because the evaluation of risk must reflect the proposed use of a device in a study.”^27^

As an example, FDA views “Menstrual Tampons (cotton or rayon only)” as nonsignificant risk medical devices unlikely to pose serious risks to participants in clinical investigations to evaluate product safety, effectiveness, or substantial equivalence to other existing products.^28^ If, however, a researcher proposed to jam these NSR devices far up into research participants’ noses as part of a basic scientific study to collect and analyze human nasal secretions, that research might pose serious safety risks despite using a device that is quite safe in its intended use.

FDA has determined that MRI devices pose “significant risk” in investigations where the device operating conditions involve a magnetic field above 8 Tesla for persons more than one month old (or 4 Tesla for neonates under that age) or if various other operating parameters are exceeded.^29^ In this same guidance, however, FDA cautions that “[t]hese criteria apply only to device operating conditions. Other aspects of the study may involve significant risks and the study, therefore, may require [FDA oversight] regardless of operating conditions.”^30^ It is completely erroneous to conclude that “MRI systems up to 8T are NSR devices”^31^ and, therefore, any research with low-field portable MRI scanners is NSR research.

Portable, low-field MRI scanners reduce but do not eliminate the safety risks of neuroimaging research, particularly in study protocols calling for repeated imaging of the same individuals to monitor changes over time or in response to different stimuli. Even when FDA or its counterparts in other nations have cleared or approved an imaging device as reasonably safe and effective for an intended clinical use, researchers may press the device into off-label uses for which its safety is unknown.

In light of these risks, scholars are concerned that portable MRI research could fall into jurisdictional gaps in the Common Rule and HIPAA Privacy Rule. The Common Rule only applies to research that is either funded by federal agencies that implement the Common Rule or carried out at academic research institutions that have voluntarily agreed to subject all of their research to the Common Rule regardless of the funding source.^32^ The HIPAA Privacy Rule applies to institutions providing health care services and to their employees, which takes in most research at academic medical centers but can leave out a great deal of commercial research and even academic research done by nonmedical departments at universities structured as HIPAA “hybrid entities.”^33^

For portable MRI research in field settings, it is possible that neither of these regulations would apply, depending on how the research is funded, where the research data are stored, and whether the investigators happen to be employees of a HIPAA-covered entity. For example, the commercially funded peanut-butter taste test hypothesized earlier seemingly could escape regulation under the HIPAA Privacy Rule and the Common Rule.

### B. Can FDA Fill the Regulatory Gap?

FDA’s research regulatory framework offers a potential backstop for oversight of portable MRI research. While it will not always apply, FDA’s framework does have a useful potential to help fill the regulatory gap when research escapes oversight under the Common Rule and HIPAA Privacy Rule.

FDA’s research regulations were designed to ensure the ethical conduct of clinical trials — often privately sponsored — that assess safety and effectiveness of investigational drugs and medical devices as a prelude to seeking FDA premarket review and clearance/approval for commercial sale. However, FDA’s research oversight can also apply to basic scientific research — that is, studies in which there is no plan to use the research results to inform regulatory decisions — if the research uses FDA-regulated medical devices in ways that pose significant risk to the research participants.^34^

When it applies, FDA’s framework requires informed consent^35^ and review by an Institutional Review Board (IRB) qualified to oversee FDA-regulated clinical studies,^36^ and in these respects it resembles the Common Rule. FDA’s regulations differ subtly from the Common Rule where informational research (research with previously stored data and biospecimens) is concerned: for example, FDA’s regulations are more cautious than the Common Rule is about allowing unconsented research use of deidentified biospecimens.^37^ FDA’s research regulations also require disclosure of financial conflicts of interest such as an investigator’s intellectual property rights in an investigational drug or device that is under study,^38^ and they require appropriate labeling, manufacturing, and distribution of investigational products to disclose their experimental nature and restrictions on their use.^39^

Where FDA’s regulations differ most starkly from the Common Rule is FDA’s Part 812 Investigational Device Exemption (IDE) regulations,^40^ which are potentially important in portable MRI research. Part 812 is more in the nature of a consumer safety regulation than a traditional research ethical framework. Exposing research participants to as-yet-unapproved, experimental medical devices during a research study directly subjects them to the risk of product-related injuries. Product safety risks also can arise when research uses an FDA-cleared or approved device but deploys it in a novel (off-label) way for which its safety is unknown. Part 812 aims to protect research participants from those risks.

Part 812 implicitly requires that a federal regulator — FDA — review the proposed research protocol in which a device will be used. The Common Rule has no corresponding requirement, instead relying on IRBs, often staffed with employees of the institution that wishes to conduct the research, to assess whether “[r]isks to subjects are minimized” and ‘[r]isks to subjects are reasonable in relation to anticipated benefits, if any, to subjects, and the importance of the knowledge that may reasonably be expected to result.”^41^ When Part 812 applies to portable MRI research, it empowers a skeptical regulator — FDA — to scrutinize the research protocol before approving a proposed research use of a device.

An important point is that FDA’s Part 812 regulations do not distinguish whether research is privately or federally funded or whether it takes place within a HIPAA-covered academic research institution or not. Any use of a device in research that is conducted anywhere by anybody can trigger oversight under Part 812, if the research use of the device poses significant risk to the research participants.^42^

Part 812 has an exemption for research with diagnostic devices,^43^ but portable MRI research does not fit in this exemption. The exemption requires all four of the following conditions to be met: (1) the testing must be non-invasive; (2) the testing must not “require an invasive sampling procedure that presents significant risk”;^44^ (3) it must not by design or intention introduce energy into a subject; and (4) the testing must not be “used as a diagnostic procedure without confirming the diagnosis by another, medically established diagnostic product or procedure.”^45^ Even if portable MRI research meets conditions (1), (2), and (4) for an exemption, it fails condition (3). This means portable MRI research is potentially subject to regulation under Part 812, if other jurisdictional conditions are met.

The crucial question, then, is when will FDA’s Part 812 IDE regulations apply to portable MRI research? FDA’s authority as a research regulator is incident to its authority to regulate medical products such as drugs and devices. Accordingly, the types of research FDA can regulate are narrower than the sweeping definition of “research”^46^ that the Common Rule regulates. As a general rule, Part 812 allows FDA to regulate investigations *of* medical devices (“clinical investigations of devices to determine safety and effectiveness”^47^), as opposed to regulating research that merely uses devices as tools to study other phenomena in nature.

Merely using a device — even one that FDA has never previously cleared or approved — as a means to study a medical or physiological phenomenon will not, by itself, cause the research to fall under Part 812. The agency’s own training materials state, as the basic rule, that an IDE is not required for “basic physiological research” that is “investigating a physiological principle” with “no intent to develop the device for marketing,” if the investigation is “only using the device to address the research question.”^48^ FDA first enunciated this principle in the preamble to the final rule when Part 812 was first implemented in 1980.^49^ However, two important exceptions to the basic rule sometimes let FDA regulate basic scientific “investigations to expand medical knowledge or conduct fundamental research.”^50^

The first exception is that a basic scientific study can fall under Part 812 if FDA determines that it implicitly incorporates a study of whether the device is safe and effective for a given use. “If the expansion of medical knowledge or the conduct of fundamental research involves an investigation to determine the safety or effectiveness of a device, an IDE will be required.”^51^ Suppose a portable MRI study does not have, as one of its aims, to prove the device is effective as a diagnostic tool for assessing learning disabilities. However, the research protocol envisions that researchers will use the device’s outputs to help them make such assessments. FDA might conclude that the study, in fact, implicitly incorporates a study to determine whether the device is effective for that use. On this basis, FDA could require an IDE.

The second exception is that FDA can regulate the use of a device, even in a basic scientific study, if the use of the device presents “significant risk” to the research subjects.^52^ In these instances, the research is not studying the device itself but using the device to study other medical or scientific phenomena in ways that pose significant risk to research participants. This might occur even with an FDA-cleared or approved portable MRI scanner, if the research uses it in ways that deviate from the intended use stated in its product labeling. FDA “approval of a drug or medical device for one intended use does not assure its safety and effectiveness for other uses.”^53^

A concrete example helps clarify the types of information that might appear in a portable MRI device’s labeling. FDA’s 510(k) database shows clearances for several evolutions of the Hyperfine Swoop® Portable MRI System™. Its “intended use” simply states that the device is “for producing images that display the internal structure of the head where full diagnostic examination is not clinically practical. When interpreted by a trained physician, these images provide information that can be useful in determining a diagnosis.”^54^

Following a practice that is not uncommon for *in vivo* diagnostic devices, this device is labeled for analytic use without making any specific clinical claims. An analytical claim merely asserts that a diagnostic device can accurately and reliably detect the presence or absence of the “analyte” — the characteristic of reality that it purports to detect.^55^ An example of an analytical claim is: “This device produces accurate images of calcification in a patient’s arteries.” A clinical claim goes farther and asserts that presence or absence of the analyte reveals clinically meaningful information about a person’s state of health.^56^ An example of a clinical claim might be: “This device is for producing images of calcification in arteries as an aid to diagnosing coronary artery disease.”

In the Hyperfine Swoop® Portable MRI System™ labeling, the intended use is simply to produce analytically valid images of the internal structure of a person’s head. The labeling ascribes no clinical meaning to that information, and any assignment of clinical significance is left to the trained physician ordering the test. The labeling also notes that it is a prescription device.^57^ Ordinarily, medical device labeling must provide adequate directions for use that would enable a *layperson* to understand both the conditions for which the device is to be used and how to use it safely and effectively.^58^ When this is not possible, for example, if the device or the information it produces is too complex for laypeople to use safely without a doctor’s involvement, FDA can exempt the device from this requirement and allow its directions to be written for healthcare professionals, but then the device becomes a prescription device.^59^

When a device is labeled as a prescription device for use pursuant to a doctor’s order, it is not a consumer product intended for over-the-counter sale to economists, lawyers, and other medical laypeople. If the device produces analytically valid results intended for interpretation by a trained physician, it would be inconsistent with the product’s labeling for an economist or lawyer to interpret the clinical significance of the images the scanner produces. Doing so could pose serious risks unless the research plan includes appropriate medical personnel to order testing and assist with interpretation of results. Device manufacturers are prohibited from marketing their products for unapproved uses, including uses that would place nonmedically trained researchers in the position of ascribing clinical significance to images the scanner produces.

When might a layperson’s research use of a portable MRI device cross the line into ascribing clinical significance to imaging results? Consider three examples:

Example #1.

A professor of fine arts uses a portable MRI scanner to capture images of volunteers’ brains, with the aim of gluing the images onto a canvas to create an artistic collage portraying the beauty of the human brain. Here, the professor is only interested in collecting analytically valid images but ascribes no clinical significance to them. Unless the scanner is used in a way that creates significant safety risks for the participants, this use would not require an IDE.

Example #2.

An educational researcher plans to use portable MRI scans of schoolchildren as an aid to studying learning disabilities. In this study, the researcher, in effect, intends to use the device “in the diagnosis of disease or other conditions [that is, learning disabilities], or in the cure, mitigation, treatment, or prevention of disease, in man or other animals.”^60^ As used in this research, the scanner meets the definition of an FDA-regulable medical device. However, FDA has not cleared the scanner for diagnostic use by medical laypersons. This unapproved use carries a risk of diagnostic error and could require an IDE.

Example #3.

Psychology and marketing researchers use portable MRI in a study exploring the relationship between brain structure and a person’s fashion choices.^61^ Bad fashion choices are not a medical “disease or condition,” so researchers will not be ascribing clinical significance to the imaging results. This study seems to be basic scientific research that would not require an IDE unless the scanner were used in a way that poses significant safety risks.

### C. The Process for Determining Whether Research Requires an IDE

The previous examples show how FDA’s IDE regulations can fill regulatory gaps for some (but not all) portable MRI research conducted by medical laypersons. When basic scientific research falls through gaps in the Common Rule and HIPAA Privacy Rule, FDA’s IDE regulations offer a final line of defense, but these regulations may not always apply. This section summarizes the process for determining whether research requires an IDE. It then explores how the advent of portable MRI is placing strains on this process, possibly making it hard for FDA to enforce its IDE regulations even when they technically apply.

Basic scientific research using portable MRI does not require an IDE unless the research incorporates a study of device safety and effectiveness or poses significant risk to research participants. The sponsor of the study must obtain an IRB approval of the investigation “after presenting the reviewing IRB with a brief explanation of why the device is not a significant risk device.”^62^

Part 812 tasks IRBs, in the first instance, with determining whether a proposed research use of a device poses significant risk.^63^ If an IRB concludes that the device poses no significant risk, it can approve the research without an IDE. FDA does, however, retain authority to second-guess the IRB’s determination and step in and require an IDE if the agency determines the device does pose significant risk in the planned research use. The question, of course, is how the FDA would know the research is happening — a question the next subpart explores.

Part 812 defines a “significant risk device” as one that (1) is an implant and presents a serious risk, or (2) is for a use in supporting or sustaining human life that presents a serious potential for risk — neither of which seems relevant to portable MRI research — but it adds a third and fourth criteria that are potentially relevant.^64^

The third criterion is that using a device in basic scientific research can pose significant risk if the research applies the device “for a use of substantial importance in diagnosing, curing, mitigating, or treating disease, or otherwise preventing the impairment of human health and presents a potential for serious risk to the health, safety, or welfare of a subject.”^65^ Using an FDA-cleared device that is intended for analytical use only (e.g., to make images of a person’s head) and assigning clinical significance to these results (e.g., that the person has a learning disability, depression, or is likely to relapse into substance abuse) could constitute significant risk and trigger the need for an IDE. This is particularly true if the research plan includes returning these results to the participants or using the results in ways that might affect the participants’ health and well-being during the research. An example of the latter would be if investigators use the results to assign participants to different arms of a clinical study that will compare two different interventions for learning disabilities. Also, if the intended use of a device envisions that trained physicians can assess the clinical significance of images, research might pose significant risk if it allows non-physician investigators (such as economists, market researchers, legal scholars, or educational researchers) to perform this role.

The fourth criterion is that a device used in basic scientific research can pose significant risk if it “otherwise presents a potential for serious risk to the health, safety, or welfare of a subject.”^66^ This language is very broad and seemingly allows IRBs to consider non-physical risks, such as the psychosocial harms of labeling a child as “small-brained.” As just discussed, returning such conclusions to the research participants, or using the results to stratify them to receive different interventions during a clinical study, might pose significant risk and require an IDE under Part 812.

Obviously, much depends on the diligence of IRBs as they review specific research protocols. Part 812 looks to IRBs to assess whether proposed research uses of portable MRI devices pose significant risk, which can exist even in basic scientific studies that rely on devices FDA has cleared or approved. For such devices, IRBs will need to scrutinize whether the proposed research use accords with the intended use, conditions for use, and warnings stated in the device’s labeling and must carefully assess the specific ways investigators plan to use and communicate the device outputs.

### D. How Portable MRI Research Disrupts the Existing Regulatory Process

Portable MRI is reshaping the landscape of neuroimaging research, engaging new and non-traditional actors (such as citizen scientists) in research. These new actors may not have an IRB (or the means to hire an independent IRB) to assist with IDE compliance. Indeed, they may not even know that FDA’s IDE regulations exist. If they use portable MRI scanners in ways that subject research participants to significant risk, FDA has jurisdiction to step in, but how would FDA know the research is occurring? What if a citizen scientist, after building an open-source MRI scanner, fails to follow FDA’s registration and listing regulations, which require medical device manufacturers to make themselves known to the agency so it will know to inspect and regulate them?^67^ Existing regulations work largely because the regulated entities cooperate in making them work.

Modern innovator nations entered the 21^st^ century with legacy regulatory frameworks that were designed for 20th century industry structures.^68^ Those old industry structures included a relatively small number of large-scale medical product manufacturers, which made it feasible and cost-effective for agencies like FDA to maintain close regulatory oversight.^69^ Medical product manufacturers were generally compliance-oriented and willing to do their part to comply with the regulations in return for the competitive protections that high regulatory barriers to entry provide.^70^ Thus protected from competition, they could achieve the scale of operations and the financial capacity to cover the costs of generating evidence to support FDA’s premarket regulatory review and post-market oversight of their products.^71^ Public funders like the NIH supported upstream basic scientific research, sited mainly at academic institutions, and the funders were able to induce voluntary compliance with research ethical and privacy norms in return for grants.^72^ Compliance-oriented product manufacturers and commercial research organizations embraced many of these same norms even when law did not require them to do so.^73^ In both the public and commercial spheres, competition for research funding was intense, and rigorous peer review processes deterred the pursuit of dubious or low-valued “junk” science.

A 2017 study by the National Academies of Science, Engineering, and Medicine identified trends that are disrupting these 20th-century biotechnology industry structures.^74^ Technologies like low-field portable and open-source MRI, direct-to-consumer diagnostics, and do-it-yourself gene editing are enabling this disruption. Agencies like FDA now have the unenviable task of trying to regulate new technologies using old regulatory frameworks designed for industry structures that the technologies themselves are undermining. The regulatory challenge is not the new technologies themselves; products like portable MRI, advanced diagnostics, and even gene-editing tools are not really all that different from products FDA has successfully regulated in the past. Instead, the major regulatory stressor comes from the new business models and industry structures that these new products make possible.

Agencies like FDA now have the unenviable task of trying to regulate new technologies using old regulatory frameworks designed for industry structures that the technologies themselves are undermining. The regulatory challenge is not the new technologies themselves; products like portable MRI, advanced diagnostics, and even gene-editing tools are not really all that different from products FDA has successfully regulated in the past. Instead, the major regulatory stressor comes from the new business models and industry structures that these new products make possible.

“The very concept of ‘regulation,’ as developed and practiced under the web of old statutes [that FDA oversight] rests on, fails unless there are suitable entities to regulate.”^75^ In their work on disruptive innovation in health care, Christenson, Grossman, and Hwang stress that new technologies merely *enable* disruptive change, but are not alone sufficient to bring it about.^76^ Real change happens only when a new technology is combined with innovative business models and new value networks.^77^ The 20th-century industry players FDA traditionally regulated are not going away, but they will now be competing alongside an array of new players with different business models (e.g., do-it-yourself brain-hacking clubs where citizen scientists scan their own brains using open-source scanners) and different funding mechanisms (e.g., crowd-funded research and dues-paying neuroscience clubs), operating at different sites (e.g., at community laboratories or in people’s homes) and in larger numbers and at smaller scales than the 20th-century medical product manufacturers for which FDA’s regulations were so well suited.

It is not the technologies, but the changes in industry structure they bring, that make regulation difficult. Technologies like portable MRI support a “democratization”^78^ of biomedical research and manufacturing, engaging large numbers of smaller players in activities previously centralized in a discrete number of large academic medical centers and monolithic industrial corporations. The regulatory challenge is similar to what Kellen Zale observes in her study of the platform-based “sharing economy,” where consumers travel in cars summoned from ride-hailing services like Uber and Lyft and sleep in beds rented from strangers they met through Airbnb.^79^ “[S]cale is a defining feature and fundamental challenge of the sharing economy” because “when everything is small, the regulatory challenge is immense.”^80^

Twentieth-century regulations sometimes excluded small players from regulation altogether.^81^ Even regulators like FDA, which has jurisdiction to regulate device manufacturers regardless of their size, often used their discretion to avoid regulating small players on various theories. These theories include, for example, that small-scale activities have only minor impacts on interstate commerce, or that it is harsh to subject small players to heavy regulatory compliance burdens, or that it would cost more than it is worth to scale up regulatory agencies to find, inspect, and regulate them.^82^ This last item, regulatory efficiency, is a major issue: a plane ticket costs the same, whether FDA is flying its inspector to a garage where a citizen scientist manufactures one open-source MRI machine, or to a large medical device manufacturer producing hundreds of machines. The budget and staffing to regulate large numbers of small-scale actors can exceed what Congress, and the public, are willing to finance.

FDA’s traditional regulatory tools, such as facility inspections, are hard to implement when the manufacturing facility is an open-source brain hacker’s home. Traditional product developers in effect consent to inspection when they accept an FDA clearance or approval to market their products, and their refusal to allow inspection is prohibited by federal law.^83^ But if FDA wishes to inspect an open-source portable MRI manufacturing operation in a private citizen’s garage, the agency first needs to obtain a search warrant.^84^

Finally, policymakers are skeptical that coercive, top-down regulation is the best way to motivate small-scale and individual players, who may not be compliance-oriented and usually lack well-staffed regulatory compliance departments to interface with regulators.^85^ Scholars theorize that social norms, community-based oversight, and voluntary codes of conduct might be more effective at promoting ethical research use of portable MRI and other emerging technologies.

Unfortunately, these latter approaches strike many policymakers and members of the public as porous and unenforceable. This criticism misses the point. It is true that voluntary ethical codes are not legally enforceable. Yet mandatory, command-and-control regulations are *equally unenforceable* if our society is unwilling to fund and staff agencies like FDA at the levels it would take to oversee large numbers of small-scale players who may be making and using portable MRI machines and other “democratizing”^86^ technologies like do-it-yourself gene editing tools. Viewed in this light, faith in mandatory regulatory frameworks is misplaced. Voluntary ethical codes and other soft-law measures, such as those Shen et al. have proposed for portable MRI,^87^ might protect research participants as well (and perhaps more effectively) than traditional regulatory frameworks can do.

## II. Do Current Regulations Protect Society’s Broader Interest in Ensuring Responsible Research Use of Portable MRI Technology?

Portable MRI scanners vastly expand scientists’ ability to collect and store data about people’s brains. A threshold question is whether amassing such data at an increased scale is a good idea: Will doing so serve the public good? A troubling aspect of the major U.S. federal research regulations is that, even when they apply, they focus heavily on the risk research poses to participating individuals but deemphasize concerns about the public benefits and harms of data collection and whether research accords with public expectations and values.

Twentieth-century research ethical frameworks largely steer clear of the latter questions under a simplifying assumption that research data collection is socially and morally acceptable as long as the participants consent to it.^88^ Yet individuals’ autonomous choices do not always produce good societal outcomes, and even consented data collection can inflict public harms. In 21st-century research involving large-scale collection and processing of data, there is growing unease that informed consent — even when it seems morally necessary — may not be morally sufficient to prevent public harms. Individuals’ privacy is interdependent.^89^ Those who consent to share their data for research can inflict negative externalities (including privacy harms) on non-participants.^90^

Bioethicists first recognized this problem in a few, seemingly narrow, contexts, such as genetic research that reveals information about participants’ non-consenting family members.^91^ Similar concerns arise in research with Indigenous peoples, where data from a few participating individuals can support inferences about other, non-consenting members of small tribal communities.^92^ As data collection grows in scale — and as studies become less biased and more generalizable (which is good for scientific progress and promotes health equity) — privacy interdependencies emerge as a broader societal problem potentially threatening everyone.^93^

### A. Societal Risks of Portable MRI Research

In today’s data-intensive research enterprise, privacy depends not just on the input data people contribute to a study, but on the inferences researchers can draw about them using other people’s data.^94^ Research, as a quest for “generalizable knowledge,”^95^ expands science’s capacity to know things about you even when you are not there. If people with whom you share similarities consent to research, the research reveals information about you, and even with the modern focus on human diversity and bias, the fact remains that humans are one species with more similarities than differences. This underlying human similarity was apparent when an AI-based breast cancer screening tool trained on data from predominantly white patients at a leading American academic medical center proved valid even when tested on diverse global patient populations.^96^ Unfortunately, scientists cannot yet predict which human characteristics are reliably shared and which ones make us unique and diverse, so careful model validation remains essential to detect potential bias.

The types of research societies choose to pursue — and the types of data collected and the scale of data collection — can affect all members of a society whether they consent to the research or not. Twenty-first-century informational research creates systemic risks, creating a need for collective ethical deliberation and societal buy-in.^97^ Widespread collection of brain data using portable MRI in a wide array of field settings is an example of this phenomenon. What is missing — and what is needed — is a “social licence”^98^ or licensing framework able to assess: (1) which research uses of portable MRI scanners are likely to benefit the public and which offer low benefits to society or raise ethical concerns; (2) “[t]he extent to which entities (public and private) are constrained to meet societal expectations” when pursuing portable MRI research, and (3) when research efforts should “avoid activities that societies deem unacceptable.”^99^

### B. The History of Social Licensing and Public Benefit Analysis in Biomedical Research

The notion that biomedical research — and in particular, the large-scale generation and use of personal health data in research — should require some sort of social license (or licence in British literature) emerged among data ethicists in the first decade of the 21st century^100^ and attracts a growing following outside the United States.^101^ The basic idea is that there needs to be community or even society-wide approval for research involving widespread creation or use of personal data, in order to ensure that the research is “beneficial, ethical, responsible, and sustainable.”^102^ The subtext of the social licensing movement is that current laws and regulations governing research uses of data, which many nations implemented after 1970, fall short of this standard: “to date, predominantly medical and scientific stakeholders have been in the position to determine the ethical boundaries of medicine, care and medical research…while at the same time, ‘lay’ and societal stakeholders have largely been left out.”^103^

Social licensing resonates with a US-based critique of the Common Rule and HIPAA Privacy Rule, which rely on “top-down, expert- and scholar-led” processes for approving which research topics to pursue and for deciding the ethical and privacy protections research participants will receive.^104^ Research funders, scientific peer reviewers, regulators, and IRBs make these decisions with little public knowledge or input.^105^ Informed consent grants people a narrowly limited “take-it-or-leave-it right to refuse to let their data be used in research if they dislike the research protocol or distrust the privacy and ethical protections that others have set for them” but they have no real voice in shaping the types of data-driven research our society pursues or the ethical and privacy protections that will apply.^106^

All too often, private commercial actors operating outside the reach of research regulations make these decisions based on commercial considerations: What might be profitable rather than what is in the public’s interests? Even when the Common Rule and HIPAA Privacy Rule apply — for example, in federally funded research conducted at HIPAA-covered academic institutions — IRB oversight is a scheme of expert-led, top-down governance. Efforts to include community representatives on IRBs and elicit broader community engagement undoubtedly help but are sometimes criticized as tokenism that fails to ensure a representative sample of public opinion.^107^ Moreover, IRBs approving data uses often include employees of the research institution that wishes to conduct the research, making them potentially conflicted, and IRBs lack basic procedural safeguards and transparency.^108^ Finally, the regulatory criteria IRBs apply when approving studies that collect and use personal data lack a public benefit requirement of the sort social licensing theorists have described.^109^

What, exactly, is a public benefit requirement? A seminal study by Carter et al. concludes that the necessary conditions for social licensing of research include: (1) reciprocity, (2) non-exploitation, and (3) service of the public good.^110^ Reciprocity and non-exploitation address various issues. For example, can people who donate their data to research feel confident that researchers will publish study results rather than hoarding discoveries as trade secrets? Does the research seek to confer social benefits (if not on the current research participants, then at least to similarly situated persons in the future)? Are the researchers who use people’s data suitably qualified to produce valid scientific results? Will data users reciprocate people’s data contributions by avoiding abusive practices such as re-identifying, re-using data for unapproved purposes, and re-sharing (or even selling) the data?^111^ Research participants have been shown to “mistrust commercial interests, especially where these might be perceived as profiteering or resulting in excess profit.”^112^

Carter et al.’s public benefit requirement requires research uses of people’s health data, particularly if done without their well-informed consent, to advance the public good — such as advancing scientific understanding generally or improving health care at a broad, national level (or at least for people similarly situated to those whose data are being processed during research) — as opposed to serving narrowly private or commercial interests.^113^ This concept is reminiscent of the “public use” requirement in U.S. takings law,^114^ and as Thomas Merrill points out in his work on takings theory, it is sometimes easier to describe what *is not* a “public use” than to describe precisely what it *is.*^115^

Carter et al. note that “the persistent problem of non-publication of study results” is a red flag that data are being used to generate intellectual property for private commercial gain, rather than to disseminate knowledge for public benefit.^116^ Even before publication of study results becomes an issue, it is a red flag if data users are non-transparent and decline to disclose the nature of their studies on public research-tracking websites like ClinicalTrials.gov.^117^ Additional red flags arise when people’s health data are used to develop new treatments to be offered at whatever-the-market-will-bear prices that few of the participating data subjects would be able to afford.^118^ The social licensing movement respects that members of the public are a reservoir of “I know it when I see it”^119^ wisdom about whether specific data uses are likely to serve the public good, yet current research oversight frameworks do a poor job of tapping into that reservoir of public wisdom.

### C. Why U.S. Research Regulations Lack a Public Benefit Requirement

In the U.S., public benefit requirements have an unfortunate history of being recommended by federal advisory bodies but not ultimately incorporated into regulations like the Common Rule and HIPAA Privacy Rule. The Common Rule does, of course, require IRBs to ensure that *all* regulated research meets the basic criterion stated at 45 C.F.R. § 46.111(a)(2), which requires that “the risks of research must be reasonable in relation to the anticipated benefits of the research — if any — to the individual and the importance of the knowledge that may reasonably be expected to result from the research.”^120^ Is that not a public benefit requirement? The answer is “no,” because of a distinction highlighted during rulemakings to develop the HIPAA Privacy Rule in 2000^121^ and 2002.^122^ A brief history helps clarify the point at issue.

Public benefit requirements were first debated in the 1970s in connection with regulatory criteria for allowing *unconsented* use of data in research. Up until the late 1970s, it had been common in the U.S. and elsewhere to use people’s medical records in biomedical research without individual consent.^123^ In that era, biomedical research was thought to pose minimal privacy risk because it usually was conducted by health care professionals at health care institutions that were already subject to strong legal duties of confidentiality under general health laws.^124^ As electronic storage and processing of health data entered the picture in the 1970s, an early set of Fair Information Practices from 1973 first proposed obtaining informed consent for research uses of people’s identifiable health data.^125^ This proposal influenced policy both in the U.S. and internationally but raised concern that the new consent requirements might block socially beneficial research or lead to biased study results.^126^

Bioethicists explored whether, and under what circumstances, unconsented data sharing might sometimes be ethically justified.^127^ For such uses to be permissible, the “central ethical issue” is to ensure that the potential public benefits of unconsented data uses are sufficient to outweigh the individual privacy interests at stake.^128^ Two U.S. federal advisory bodies working late in the 1970s determined that unconsented uses of data in research should be allowed only if “the importance of the research or statistical purpose for which any use of disclosure is to be made is such as to warrant the risk to the individual from additional exposure of the record or information contained therein,” and if an IRB determines that this condition is met.^129^ That was, in effect, a public benefit requirement for using data in research when well-informed consent is not possible.

Unfortunately, this public benefit requirement was never incorporated in the criteria for IRB/Privacy Board approval of unconsented data uses under the waiver provisions of the Common Rule and HIPAA Privacy Rule.^130^ The long history of how this happened is outlined elsewhere.^131^ Of concern here is that the U.S. Department of Health and Human Services (HHS) did propose to include the advisory bodies’ recommended public benefit requirement in the HIPAA Privacy Rule’s waiver provision^132^ but received “a large number”^133^ of negative public comments. Many of these came from IRBs, warning that they felt unqualified to balance the public and private interests at stake and to make consistent value judgments about which lines of research are important to society.^134^

In response, HHS revised the public benefit requirement in 2000, making it identical to the basic criterion IRBs already apply under 45 C.F.R. 46.111(a)(2). This was problematic, however, because the criterion in 45 C.F.R. 46.111(a)(2) is a minimal threshold intended to screen out research that is so lacking in scientific merit that it would be unethical to proceed with the research even if people consent to it.^135^ Having IRBs use this criterion to approve waivers for *unconsented* data uses made no sense: it implied that in any research where consent can ethically be allowed, it can also be waived. To correct this conceptual error, HHS deleted the controversial public benefit requirement from HIPAA’s waiver provision in 2002.^136^

### D. Who Should Decide Whether Research Offers Public Benefits?

The Common Rule and HIPAA Privacy Rule, even when they apply to portable MRI research activities, require no public benefit analysis of the sort social licensing advocates call for.^137^ The fact that IRBs feel unqualified to apply a public benefit requirement^138^ poses a genuine problem: if IRBs will not do it, federal agencies cannot do it. Having state actors suppress portable MRI research that they consider misguided, ethically questionable, or of low scientific value would raise serious First Amendment concerns.

Proponents of expansive governmental regulation to address the perceived dangers of research sometimes gloss over how tricky it is to steer the direction of research without violating the freedom of scientific inquiry. This freedom is essential to the advancement of science, which moves forward in fits and starts as old theories fail to explain observed phenomena and are replaced by new explanatory paradigms.^139^ “[S]cientific knowledge is often evaluated based on probabilistic theories, not on a bright line around ‘truth.’”^140^ Research that appears misguided or scientifically dubious today may produce tomorrow’s consensus truth.

Apart from its instrumental value to the advancement of science, the freedom of scientific inquiry enjoys potential First Amendment protections, which constrain governmental power to dictate the topics researchers can and cannot study. While “there is no specifically enumerated right to research in the U.S. Constitution, certain commentators argue that support for such a right could be derived from the Fourteenth Amendment right to personal liberty and the First Amendment right to free speech.”^141^ Research produces findings that serve as inputs to speech, and thus “the First Amendment must also be concerned with the production of ideas and information.”^142^

Modern First Amendment doctrine emerged fairly recently (in the 1920s and 30s) after Justice Holmes’ 1919 *Abrams* dissent^143^ and a series of 1920–27 dissents and concurring opinions^144^ from Justice Brandeis.^145^ Observers of U.S. research policy sometimes forget how profoundly its emergence shaped 20th-century research oversight in the United States. In the 1940s, President Roosevelt’s vision of extending wartime research funding into a post-war program of public support for scientific research gave birth to today’s National Institutes of Health (NIH) and National Science Foundation (NSF) but raised concerns that the government’s power of the purse might unduly interfere with freedom of scientific inquiry.^146^ To allay these concerns, the NIH adopted the use of “study sections”^147^ (peer-review groups of private biomedical scientists), rather than government officials, to prioritize research proposals for federal funding.^148^

Later, concerns about scientific freedom shaped the decision to involve private IRBs in federal research oversight.^149^ In the 1970s, Congress empowered the National Commission for the Protection of Human Subjects of Biomedical and Behavioral Research to design the Common Rule.^150^ This Commission concluded that “if a case arose,” the freedom of scientific inquiry would likely receive First Amendment protection.^151^ The Commission felt, however, that private research institutions “may empower the IRB to apply both content and manner restrictions” on their personnel as conditions of employment and for receipt of research funds, “whether or not such a system would be constitutional if directly imposed by the state on nonfunded research.”^152^

The role of IRBs in U.S. research regulation was, at its inception, a First Amendment work-around: a strategy of relying on private actors to regulate the content and manner of research in ways that federal agencies, facing constitutional constraints, seemingly could not do. If IRBs feel unqualified, and if regulatory agencies are constitutionally unable to impose public benefit requirements on portable MRI research, then who is left to do so? What remains are various “private ordering” solutions that mobilize private actors to set boundaries for permissible use of portable MRI scanners in field research.^153^

These solutions could include, for example, a voluntary Code of Conduct developed by neuroscientists and ethicists, coupled with agreements by scholarly journals not to publish results of studies that violate the Code, or using the power of the press (or of social media) to cast light on abusive or questionable research practices and mobilize the public “cancellation” of those who engage in dubious research practices.

Unfortunately, such solutions often replicate the same top-down, expert- and scholar-led decision structures seen in governmental regulatory schemes. It often proves easier to convene a panel of eminent neuroscientists and ethics experts to draft a Code of Conduct than to survey a representative sample of a fractious public and build consensus on the privacy and ethical protections people really want. Social licensing implies doing the messy work of tapping into the vast reservoir of public wisdom about which uses of portable MRI technology offer public benefits and which should be avoided. Creating appropriate institutions for this sort of public oversight is still in its early phases.

Community engagement efforts mark a step in the right direction. However, as currently implemented, these efforts sometimes engage only small, non-representative samples of affected communities and may fail to engage people on matters of greatest concern to them. Windsor et al. note that community-engaged research (CEnR) “has experienced substantial growth in the United States in recent decades, particularly in the public health arena”^154^ but cited examples in which community engagement addressed relatively minor side issues, such as the format of informed consent documents. One of the reported CEnR efforts “shortened informed consent forms by one page by incorporating feedback from community focus groups” while another led to “supplemental videos explaining the study” and “bullet points summarizing study risks and activities in clear language.”^155^ Windsor et al. gave no examples of CEnR efforts which engaged communities on major substantive issues such as, “What types of research does the community regard as socially beneficial, and what privacy protections would they like to receive?”

Unfortunately, bioethicists and IRBs continue clinging to top-down, expert-led decision processes and small, nonrepresentative focus groups. It is time for ethicists to embrace already-available digital technologies to engage affected communities in reciprocal dialogues — in real time and at scale —to guide research policies for emerging technologies like portable MRI.

These substantive decisions, all too often, continue being made by investigators, research institutions, funders, ethicists, and IRBs — in other words, through top-down, expert-led decision processes. Communities are engaged on minor points of style and format such as, “Would you rather find out what the experts already decided by reading a consent form or by watching a video?” Carter et al. caution against these “narrowly focused public relations exercises that seek to ‘capture’ the public, that is, to persuade the public of the legitimacy of decisions already taken by experts.”^156^

Social licensing, in contrast, requires reciprocal communications that grant the public a meaningful voice while decisions are still being made about appropriate data uses and privacy policies. Work is now just getting underway to harness digital deliberation tools to enable bioethicists to “hear directly and at scale from the public they are trying to protect.”^157^ These tools already are in wide use by governments and non-profit institutions around the world to engage large swathes of the public in policymaking.^158^ Digital democracy tools, such as the open-source Polis tool, incorporate advanced algorithms “for gathering, analyzing, and understanding what large groups of people think in their own words, enabled by advanced statistics and machine learning” to help identify points of emerging consensus and guide deliberations in directions that amplify it.^159^

These tools have been in use for over a decade in governmental policy-making efforts.^160^ Unfortunately, bioethicists and IRBs continue clinging to top-down, expert-led decision processes and small, nonrepresentative focus groups. It is time for ethicists to embrace already-available digital technologies to engage affected communities in reciprocal dialogues — in real time and at scale — to guide research policies for emerging technologies like portable MRI.^161^ Only by giving the public a meaningful voice in policy-setting can ethicists and research participants “co-creat[e] what is considered trustworthy” and achieve social license.^162^

## Conclusion

Field research with portable MRI has the potential to fall into gaps in major U.S. regulations such as the Common Rule and HIPAA Privacy Rule, leaving research participants with inadequate ethical and privacy protections. FDA’s Part 812 IDE requirements cover some (but not all) basic scientific research using portable MRI. This fact, and the at-times-cumbersome IRB review process to decide whether an IDE is required, may help deter inappropriate uses of portable MRI in nonmedical research.

In theory, medical laypeople like lawyers, market researchers, philosophers, and sociologists could use FDA-cleared devices off-label for a wide variety of research projects, but in many instances, they would first need to obtain an IDE. Preparing an IDE application can entail thousands of pages of documentation and delay research by many months, which could discourage ethically questionable uses of portable MRI scanners for low-valued scientific endeavors. However, portable MRI invites research activities by a diverse array of new actors, many operating at small scales or using novel business models that thwart effective regulatory enforcement, even when agencies like FDA have jurisdiction to regulate. Because of this enforcement challenge, voluntary codes of conduct and other soft-law approaches, including ideas Shen et al. proposed elsewhere in this issue, may prove as effective as mandatory regulatory approaches in this research environment.

Even when they apply, existing research regulations were never designed to protect society’s broader interest in ensuring that portable MRI research will benefit the public and accord with public values and expectations. The absence of public benefit requirements in U.S. research regulations reflects an implicit assumption that may have been true in the last century when regulations like the Common Rule, HIPAA Privacy Rule, and FDA IDE regulations were conceived, but which is no longer valid today. Specifically, policymakers assumed that research uses of highly personal data posed no privacy or psychosocial risks to individuals unless their personal data were used as inputs to the research. As long as individuals had a right of consent that let them opt out of research, their rights would not be affected, or so the reasoning went. Thus, IRBs had no need to balance the public benefits of research against the risks it might pose to non-consenting individuals, and consenting individuals could appraise the merits of the research for themselves.

This assumption no longer holds true in a modern research enterprise capable of generating and processing large volumes of personal information to produce generalizable insights that can undermine the rights of, or stigmatize, non-participating individuals, whole communities, or even society at large. The potential for field-based portable MRI research to produce ethically questionable, scientifically dubious findings that stigmatize individuals (or whole population subgroups) is just one small edge case in a larger research ethical problem. Twentieth-century, autonomy-based research ethical frameworks from the past are not up to the challenge of aligning today’s data-intensive research activities with the interests, expectations, and aspirations of affected communities, which include all of us.

Social licensing frameworks are attractive but remain in the early conceptual phase. Implementing them is the unaddressed challenge of our time. The advent of field research with portable MRI offers a timely opportunity to engage the public, actively and at scale, in co-creating research policies to promote responsible, ethical use of this promising new technology.

## References

[1] F.X. Shen et al., “Emerging Ethical Issues Raised by Highly Portable MRI Research in Remote and Resource-limited International Settings,” Neuroimage 238 (2021): 118210; F.X. Shen et al., “Conducting Research with Highly Portable MRI in Community Settings: A Starter Guide to Navigating Ethical Issues and ELSI Checklist,” *Journal of Law, Medicine & Ethics* 52, no. 4 (2024): 767–783. 34062266 10.1016/j.neuroimage.2021.118210 PMC8382487

[2] F.X. Shen et al., “Ethical, Legal, and Policy Challenges in Field-based Neuroimaging Research Using Emerging Portable MRI Technologies: Guidance for Investigators and for Oversight,” Journal of Law & Biosciences 11, no. 1 (2024): lsae008, 10.1093/jlb/lsae008. PMC11157461 38855036

[3] *Id.* at 3 (citations omitted).

[4] *Id.* at 15.

[5] A. Cho , “MRI for All: Portable Low-Field Scanners Could Revolutionize Medical Imaging in Nations Rich and Poor—If Doctors Embrace Them,” Science 379, no. 6634 (2023): 748, https://www.science.org/content/article/mri-all-cheap-portable-scanners-aim-revolutionize-medical-imaging (last visited June 12, 2024).36821662

[6] See Shen et al. (2024), *supra* note 1, at 771.

[7] See Shen et al., *supra* note 2, at 29.

[8] See, e.g., I.G. Cohen et al., eds. *Consumer Genetic Technologies: Ethical and Legal Considerations* (Cambridge University Press, 2021); National Academies of Science, Engineering, and Medicine, *Preparing for Future Products of Biotechnology* (National Academies Press, 2017), https://www.nap.edu/catalog/24605/preparing-for-future-products-of-biotechnology (last visited June 19, 2024); L.C. Ikemoto, “DIY Bio: Hacking Life in Biotech’s Backyard,” U.C. Davis Law Review 51, no. 2 (2017): 539–568; B.J. Evans, “Minding the Gaps in Regulation of Do-it-Yourself Biotechnology,” *DePaul Journal of Health Care Law* 21, no. 3 (2020): 1–18, https://via.library.depaul.edu/cgi/viewcontent.cgi?article=1380&context=jhcl (last visited June 19, 2024).

[9] See Evans, *supra* note 8, at 11–13.

[10] See Shen et al., *supra* note 1, at 771.

[11] See National Academies of Science, Engineering, and Medicine, *supra* note 8, at 33–36; Evans, *supra* note 8, at 8–9.

[12] See Shen et al., *supra* note 2, at 6–7 (Table 2).

[13] *Id.*

[14] S.C.L. Deoni et al., “Accessible Pediatric Neuroimaging Using a Low Field Strength MRI Scanner,” Neuroimage 258 (2021): 118273, 10.1016/j.neuroimage.2021.118273. 34146712

[15] C. Koch , “Does Brain Size Matter?,” Scientific American 27, no. 1 (2016): 22, https://www.scientificamerican.com/article/does-brain-size-matter1/ (last visited June 1, 2024).

[16] Basic HHS Policy for Protection of Human Research Subjects, 45 C.F.R. §§ 46.101–46.124 (2017).

[17] Health Insurance Portability and Accountability Act of 1996, Pub. L. No. 104–191, 110 Stat. 1936 (1996) (codified as amended at 18, 26, 29 and 42 U.S.C.).

[18] 45 C.F.R. §§ 160, 164.

[19] See discussion *infra* this Part.

[20] C.G. Schwarz et al., “Changing the Face of Neuroimaging Research: Comparing a New MRI De-Facing Technique with Popular Alternatives,” NeuroImage 231 (2021): 117845, 10.1016/j.neuroimage.2021.117845. 33582276 PMC8154695

[21] B.J. Evans , “The HIPAA Privacy Rule at Age 25: Privacy for Equitable AI,” Florida State University Law Review 50, no. 741 (2023): 741–810. PMC13327739 42395654

[22] See Shen et al. (2024), *supra* note 1, at 774.

[23] “In Vitro Diagnostics” U.S. Food & Drug Administration, February 23, 2023, https://www.fda.gov/medical-devices/products-and-medical-procedures/in-vitro-diagnostics (last visited June 1, 2024); *Cf.* “In Vivo Testing Methods,” Drug Development and Diagnostics, https://drugdevelopment.fi/diagnostics/in-vivo/ (last visited June 1, 2024).

[24] See Shen et al. (2024), *supra* note 1, at 770.

[25] M.N. Hoff et al., “Safety Considerations of 7-T MRI in Clinical Practice,” Radiology 292, no. 3 (2019): 509–518. 31310177 10.1148/radiol.2019182742

[26] *Information Sheet Guidance For IRBs, Clinical Investigators, and Sponsors: Significant Risk and Nonsignificant Risk Medical Device Studies* (US Department of Health and Human Services, Food and Drug Administration, Center for Devices and Radiological Health (CDRH), January 2006), at 6, https://www.fda.gov/media/75459/download (last visited June 1, 2024).

[27] *Id.* at 8.

[28] *Id.* at 9.

[29] *Criteria for Significant Risk Investigations of Magnetic Resonance Diagnostic Devices: Guidance for Industry and Food and Drug Administration Staff* (US Department of Health and Human Services, Food and Drug Administration, Center for Devices and Radiological Health, June 20, 2014), https://www.fda.gov/files/medical%20devices/published/Criteria-for-Significant-Risk-Investigations-of-Magnetic-Resonance-Diagnostic-Devices---Guidance-for-Industry-and-Food-and-Drug-Administration-Staff-%28PDF%29.pdf (last visited July 1, 2024).

[30] *Id.* at 3.

[31] See Shen et al., *supra* note 2, at 26.

[32] 45 C.F.R. § 46.101(a); “Is All Human Research Regulated?,” U.S. Department of Health & Human Services, October 23, 2023, https://www.hhs.gov/ohrp/education-and-outreach/about-research-participation/protecting-research-volunteers/other-research/index.html (last visited June 1, 2024).

[33] See “When Does a Covered Entity Have Discretion to Determine Whether a Research Component of the Entity is Part of Their Covered Functions, and Therefore, Subject to the HIPAA Privacy Rule?” US Department of Health & Human Services, January 9, 202.3., https://www.hhs.gov/hipaa/for-professionals/faq/315/when-does-a-covered-entity-have-discretion-to-determine-covered-functions/index.html (last visited June 1, 2024).

[34] See discussion *infra* this Part.

[35] 21 C.F.R. pt. 50 (2015).

[36] *Id.* pt. 56.

[37] See generally B.J. Evans and E.M. Meslin , “Encouraging Translational Research Through Harmonization of FDA and Common Rule Informed Consent Requirements for Research with Banked Specimens,” Journal of Legal Medicine 27, no. 2 (2006): 119–166. 16728351 10.1080/01947640600716366

[38] 21 C.F.R. pt. 54.

[39] *Id.* pt. 809.

[40] *Id.* pt. 812.

[41] 45 C.F.R. §§ 46.111(a)(1), 46.111(a)(2).

[42] 21 C.F.R. § 812.2(b).

[43] *Id.* § 812.2(c)(3).

[44] *Id.* § 812.2(c)(3)(iii).

[45] *Id.* § 812.2(c)(3)(iv).

[46] 45 C.F.R. § 46.102(l).

[47] 21 C.F.R. § 812.2 (a).

[48] B.J. Evans , “The Limits of FDA’s Authority to Regulate Clinical Research Involving High-Throughput DNA Sequencing,” Food & Drug Law Journal 70, no. 2 (2015): 259–287, at 263–264 (citing L. Henley, “Clinical Investigator Training Course: How to Put Together an IDE Application,” Center for Devices & Radiological Health, Food & Drug Administration, November 14, 2013, at 17). 26302600 PMC4576719

[49] Medical Devices; Procedures for Investigational Device Exemptions, 45 Fed. Reg. 3732, at 3735 (Jan. 18, 1980). 10307350

[50] *Id.*

[51] *Id.*

[52] *Id.* at 3738.

[53] “Revised Draft Guidance for Industry: Distributing Scientific and Medical Publications on Unapproved New Uses—Recommended Practices” U.S. Food & Drug Administration, 2-3 (2014), https://www.fdanews.com/ext/resources/files/02/02-28-14-Off-LabelGuidance.pdf (last visited August 22, 2024).

[54] See Letter from Thalia T. Mills, Director, Division of Radiological Health, Cener for Devices and Radiological Health, to Christine Kupchick, Senior Regulatory Specialist, Hyperfine, Inc. (July 7, 2021), https://www.accessdata.fda.gov/cdrh_docs/pdf21/K211818.pdf (last visited June 1, 2024); Letter from Michael D. O’Hara, Deputy Director, Division of Radiological Imaging and Radiation Therapy Devices, Ctr. for Devices and Radiological Health, to Christine Kupchick, Senior Regulatory Specialist, Hyperfine, Inc. (June 10, 2022), https://www.accessdata.fda.gov/cdrh_docs/pdf22/K221393.pdf (last visited June 1, 2024); Letter from Michael D. O’Hara, Deputy Director, Division of Radiological Imaging and Radiation Therapy Devices, Center for Devices and Radiological Health, to Christine Kupchick, Senior Regulatory Specialist, Hyperfine, Inc. (July 28, 2022), https://www.accessdata.fda.gov/cdrh_docs/pdf22/K221923.pdf (last visited June 1, 2024); Letter from Thalia T. Mills, Director, Division of Radiological Health, Center for Devices and Radiological Health, to Christine Kupchick, Senior Regulatory Specialist, Hyperfine, Inc. (November 17, 2021), https://www.accessdata.fda.gov/cdrh_docs/pdf21/K212456.pdf (last visited August 26, 2024); Letter from Daniel M. Krainak, Assistant Director, Magnetic Resonance and Nuclear Medicine Team, Center for Devices and Radiological Health, to Christine Kupchick, Senior Regulatory Specialist, Hyperfine, Inc. (December 6, 2022), https://www.accessdata.fda.gov/cdrh_docs/pdf22/K223247.pdf (last visited June 1, 2024); Letter from Daniel M. Krainak, Assistant Director, Magnetic Resonance and Nuclear Medicine Team, Center for Devices and Radiological Health, to Christine Kupchick, Senior Regulatory Specialist, Hyperfine, Inc. (February 22, 2023), https://www.accessdata.fda.gov/cdrh_docs/pdf23/K230208.pdf (last visited June 1, 2024); Letter from Daniel M. Krainak, Assistant Director, Division of Radiological Imaging and Radiation Therapy Devices, Center for Devices and Radiological Health, to Christine Kupchick, Senior Regulatory Specialist, Hyperfine, Inc. (October 6, 2023), https://www.accessdata.fda.gov/cdrh_docs/pdf23/K232760.pdf (last visited August 26, 2024).

[55] See *Enhancing the Oversight of Genetic Tests: Recommendations of the SACGT* (2000), Secretary’s Advisory Committee on Genetic Testing, National Institutes of Health, at 15, note 10 (defining analytic validity as how well a test detects, identifies, calculates, or analyzes the presence or absence of the particular physical characteristic it is designed to detect).

[56] See *Id.* at 15, note 11 (defining clinical validity as how strongly the measured characteristic is correlated with the presence, absence, or risk of a specific disease or other health condition).

[57] See *supra* note 54 (providing “Indications for Use” as the first attachment to each of the cited letters).

[58] Institute of Medicine, Medical Devices and the Public’s Health: The FDA 510(k) Clearance Process at 35 Years (The National Academies Press, 2011), at 44–45, https://nap.nationalacademies.org/read/13150/chapter/1 (last visited July 14, 2024).

[59] 21 U.S.C. § 352(f); 21 C.F.R. § 801.109.

[60] 21 U.S.C. § 321(h).

[61] See Shen et al., *supra* note 2, at 3 (discussing neuromarketing applications).

[62] 21 C.F.R § 812.2(b)(1)(ii).

[63] *Id*. §§ 812.2(b)(1)(ii), 812.66.

[64] *Id.* § 812.3(m).

[65] *Id.* § 812.3(m)(3).

[66] *Id.* § 812.3(m)(4).

[67] See, e.g., “How to Register and List,” U.S. Food & Drug Administration, August 23, 2018, https://www.fda.gov/medical-devices/device-registration-and-listing/how-register-and-list (last visited June 1, 2024) (explaining FDA’s registration and listing requirements for device manufacturers).

[68] See National Academies of Science, Engineering, and Medicine, *supra* note 8, at 20–25.

[69] See K. Zale , “When Everything is Small: The Regulatory Challenge of Scale in Sharing Economy,” San Diego Law Review 53, no. 949 (2016): 964–966 (reviewing literature addressing the relationship between the scale of regulated enterprises and the efficiency and cost-effectiveness of regulatory efforts).

[70] A. Baciu et al. eds., The Future of Drug Safety: Promoting and Protecting the Health of the Public (Washington, DC: The National Academies Press, 2007).

[71] *Id.*

[72] See National Academies of Science, Engineering, and Medicine, *supra* note 8, at 69–70 (discussing the role of NIH guidelines in governance of both public and privately funded genomic research).

[73] *Id.*

[74] *Id.* at 27–58.

[75] B.J. Evans , “Programming Our Genomes, Programming Ourselves: The Moral and Regulatory Challenge of Regulating Do-It-Yourself Gene Editing,” in: I.G. Cohen et al., eds., Consumer Genetic Technologies: Ethical and Legal Considerations (Cambridge University Press, 2021): 129–145, at 131.

[76] C.M. Christenson , J.H. Grossman , and J. Hwang , The Innovator’s Prescription: A Disruptive Solution for Health Care (McGraw-Hill Education, 2008).

[77] *Id.*

[78] See Ikemoto, *supra* note 8 (discussing citizen science and the democratization of science).

[79] See Zale, *supra* note 69.

[80] *Id.* at 950.

[81] See Zale, *supra* note 69.

[82] *Id.* at 950.

[83] 21 U.S.C. § 331(f).

[84] See U.S. Constitution, amendment IV.

[85] See M. Mehlman , “Governing Nontraditional Gene Editing,” in: I.G. Cohen et al., eds., Consumer Genetic Technologies: Ethical and Legal Considerations (Cambridge University Press, 2021): 145–156, at 155–156; National Academies of Science, Engineering, and Medicine, *supra* note 8, at 36–37, 73.

[86] See Ikemoto, *supra* note 8 (discussing democratization of genetic technology).

[87] See Shen et al. (2024), *supra* note 1, at 772, 777.

[88] See discussion *supra* Part I (discussing 20th century research regulations).

[89] G. Biczók and P.H. Chia, *Interdependent Privacy: Let Me Share Your Data*, (International Conference on Financial Cryptography and Data Security, 2013), https://perma.cc/WWP2-9VC8 (last visited June 1, 2024).

[90] B.J. Evans , “Rules for Robots, and Why Medical AI Breaks Them,” Journal of Law & The Biosciences 10, no. 1 (2023): lsad001, 10.1093/jlb/lsad001. 36815975 PMC9934949

[91] M.K. Tayeh et al., “The Designated Record Set for Clinical Genetic and Genomic Testing: A Points to Consider Statement of the American College of Medical Genetics and Genomics (ACMG),” Genetics in Medicine 25, no. 3 (2023): 100342, 10.1016/j.gim.2022.11.010. 36547466

[92] K.S. Tsosie , J.M. Yracheta and D. Dickenson , “Overvaluing Individual Consent Ignores Risks to Tribal Participants,” Nature Reviews Genetics 20 (2019): 497–498. 10.1038/s41576-019-0161-z PMC7250136 31308520

[93] See Evans, *supra* note 90, at 8–9.

[94] *Id.*

[95] 45 C.F.R. § 46.102(l).

[96] A. Vala et al., “Multi-Institutional Validation of a Mammography-Based Breast Cancer Risk Model,” Journal of Clinical Oncology 40, no. 16 (2022): 1732–1740; A. Vala et al., “Toward Robust Mammography-Based Models for Breast Cancer Risk,” *Science Translational Medicine* 13, no. 578 (2021): eaba4373, 10.1126/scitranslmed.aba4373. 34767469 PMC9148689

[97] B.J. Evans and A. Bihorac , “Co-creating Consent for Data Use — AI-Powered Ethics for Biomedical AI,” NEJM AI 1, no. 7 (2024): 1–5. 10.1056/aipc2400237 PMC12412891 40918051

[98] P. Carter et al., “The Social Licence for Research: Why Care.Data Ran into Trouble,” Journal of Medical Ethics 41, no. 5 (2015): 404–409. 25617016 10.1136/medethics-2014-102374 PMC4431337

[99] S.H.A. Muller et al., “The Social Licence for Data-Intensive Health Research: Towards Co-creation, Public Value, and Trust,” BMC Medical Ethics 22, no. 110 (2021): 110, at 112 (Table 1) (citing M. Krahe et al., “Personal Health Information in Research: Perceived Risk, Trustworthiness, and Opinions from Patients Attending a Tertiary Healthcare Facility,” *Journal of Biomedical Informatics* 95 (2019): 103222). 31176040 10.1016/j.jbi.2019.103222

[100] M. Dixon-Woods and R.E. Ashcroft , “Regulation and Social Licence for Medical Research,” Medicine, Health Care, and Philosophy 11, no. 4 (2008): 381–391. 18633729 10.1007/s11019-008-9152-0

[101] See Carter et al., *supra* note 98; Muller et al., *supra* note 99; Krahe et al., *supra* note 99; E. Ford et al., “Our Data, Our Society, Our Health: A Vision for Inclusive and Transparent Health Data Science in the United Kingdom and Beyond,” Learning Health Systems 3, no. 3 (2019): e10191, 10.1002/lrh2.10191; J. Allen et al., “The Role of Data Custodians in Establishing and Maintaining Social Licence for Health Research,” *Bioethics* 33, no. 4 (2019): 502–510; P.A. Paprica et al., “Social Licence and the General Public’s Attitudes Toward Research Based on Linked Administrative Health Data: A Qualitative Study,” *Canadian Medical Association Journal Open* 7, no. 1 (2019): e40–e46; V. Xafis et al., “An Ethics Framework for Big Data in Health and Research,” *Asian Bioethics Review* 11, no. 3 (2019): 227–254; A. Ballantyne et al., “Big Data and Public-Private Partnerships in Healthcare and Research,” *Bioethics* 33 (2019): 315–326; J.A. Shaw et al., “Social License for the Use of Big Data in the COVID-19 Era,” *NPJ Digital Medicine* 3, no. 128 (2020): 1–3; N. Stephenson et al., “Health and Public Sector Data Sharing Requires Social Licence Negotiations,” *Australian and New Zealand Journal of Public Health* 46, no. 4 (2022): 426–428. 35679055

[102] See Muller et al., *supra* note 99, at 4.

[103] *Id.*

[104] B.J. Evans , “Power to the People: Data Citizens in the Age of Precision Medicine,” Vanderbilt Journal of Entertainment and Technology Law 19, no. 2 (2017): 243–265, at 255. 29118898 PMC5673282

[105] See Evans and Bihorac, *supra* note 97, at 2.

[106] *Id.* at 2–3.

[107] *Id.* at 3.

[108] C.H. Coleman , “Rationalizing Risk Assessment in Human Subject Research,” Arizona Law Review 46, no. 1 (2017): 1–51, at 13–17. 16189913

[109] See Evans, *supra* note 21, at 805–807.

[110] See Carter et al., *supra* note 98, at 408.

[111] *Id.* at 407.

[112] *Id.*

[113] *Id.*

[114] See R.P. Malloy and J.C. Smith , “Private Property, Community Development, and Eminent Domain,” in R.P. Malloy ed., Private Property, Community Development, and Eminent Domain (London: Routledge, 2008): 1–14, at 8 (discussing the public use requirement).

[115] See T.W. Merrill , “The Economics of Public Use,” Cornell Law Review 72, no. 1 (1986): 61–116, at 90–92.

[116] See Carter et al., *supra* note 98, at 407.

[117] “ClinicalTrials.gov,” U.S. National Library of Medicine, https://clinicaltrials.gov/ (last visited June 1, 2024).

[118] B.J. Evans and E.M. Meslin , “Biospecimens, Commercial Research, and the Elusive Public Benefit Standard,” in: H.F. Lynch et al. eds., Specimen Science (Cambridge, MA: MIT Press, 2017): 107–124.

[119] *Jacobellis v. Ohio*, 378 U.S. 184, 197 (1964) (Stewart, J., *concurring*) (discussing the definition of obscenity).

[120] 45 C.F.R. § 46.111(a)(2).

[121] Standards for Privacy of Individually Identifiable Health Information, 65 Fed. Reg. 82462, 82463–82466, 82697–82698 (Dec. 28, 2000).

[122] Standards for Privacy of Individually Identifiable Health Information, 67 Fed. Reg. 53182, 53270 (Aug. 14, 2002). 12180470

[123] *Personal Privacy in an Information Society* (The Privacy Protection Study Commission, July 12, 1977): 1–639, at 280, https://www.ojp.gov/pdffiles1/Digitization/49602NCJRS.pdf (last visited June 1, 2024).

[124] See Evans, *supra* note 21, at 752–753 (discussing state health laws in the US); see Carter et al., *supra* note 98, at 407 (discussing similar requirements in England).

[125] *Records, Computers, and the Rights of Citizens* (Secretary’s Advisory Committee on Automated Personal Data Systems, U.S. Department of Health, Education & Welfare, June 25, 1973), https://www.justice.gov/opcl/docs/rec-com-rights.pdf (last visited on August 26, 2024).

[126] S.J. Nass, L.A. Levit, and L.O. Gostin eds., *Beyond the HIPAA Privacy Rule: Enhancing Privacy, Improving Health Through Research*, Committee on Health Research and the Privacy of Health Information, Institute of Medicine (2009), at 209–214, http://www.nap.edu/catalog/12458.html (last visited June 1, 2024). 20662116

[127] See The Privacy Protection Study Commission, *supra* note 123, at 281, 597; *Protection of Human Subjects: Institutional Review Boards: Report and Recommendations of the National Commission for the Protection of Human Subjects of Biomedical and Behavioral Research*, (U.S. Department of Health, Education & Welfare, November 30, 1978), 43 Fed. Reg. 56174 [hereinafter cited as Protection of Human Subjects]. 10239683

[128] D. Casarett et al., “Bioethical Issues in Pharmacoepidemiologic Research,” in: B.L. Strom ed., Pharmacoepidemiology (West Sussex: Wiley, 4th ed. 2005): 587–598, at 597; *Ethical and Policy Issues in Research Involving Human Participants, Volume 1* (National Bioethics Advisory Commission, August 2001), at xviii, 103–104; P.D. Jacobson, “Medical Records and HIPAA: Is It Too Late to Protect Privacy?,” *Minnesota Law Review* 86, no. 6 (2002): 1497–1514. 15174437

[129] See *supra* note 127.

[130] See 45 C.F.R. § 46.116(f) (“Common Rule”); 45 C.F.R. § 164.512(i) (“HIPAA Privacy Rule”).

[131] See B.J. Evans , “Much Ado About Data Ownership,” Harvard Journal of Law & Technology 25, no. 1 (2011): 69–130, at 120–124.

[132] Standards for Privacy of Individually Identifiable Health Information, 65 Fed. Reg. 82462, 82463–82466, 82697–82698 (Dec. 28, 2000).

[133] *Id.* at 82698.

[134] *Id.*

[135] See Protection of Human Subjects, *supra* note 127.

[136] 67 Fed. Reg. 53182, 53270 (Aug. 14, 2002).

[137] See Carter et al., *supra* note 98, at 408.

[138] 65 Fed. Reg. 82697–82698.

[139] T.S. Kuhn , The Structure of Scientific Revolutions (University of Chicago Press, 1962).

[140] J. Blocher , “Free Speech and Justified True Belief,” Harvard Law Review 133, no. 2 (2019): 439–496, at 485.

[141] L.B. Andrews , “Is There a Right to Clone? Constitutional Challenges to Bans on Human Cloning,” Harvard Journal of Law & Technology 11, no. 3 (1998): 643–681, at 661 (footnote call number omitted). 12731552

[142] N. Ram , “Science as Speech,” Iowa Law Review 102, no. 3 (2017): 1187–1237, at 1198.

[143] *Abrams v. United States*, 250 U.S. 616, 627 (1919) (Holmes, J., *dissenting*).

[144] *Shaefer v. United States*, 251 U.S. 466, 482 (1920) (Brandeis, J., *dissenting*); *Pierce v. United States*, 252 U.S. 239, 253 (1920) (Brandeis, J., *dissenting*); *Gilbert v. Minnesota*, 254 U.S. 325, 334 (1920) (Brandeis, J., *dissenting*); *United States ex rel. Milwaukee Soc. Democratic Publ’g Co. v. Burleson*, 255 U.S. 407, 417 (1921) (Brandeis, J., *dissenting*); *Whitney v. California*, 274 U.S. 357, 372 (1927) (Brandeis, J., *concurring*).

[145] See generally D.M. Rabban , “The Emergence of Modern First Amendment Doctrine,” University of Chicago Law Review 50, no. 4 (1983): 1205–1355.

[146] D.S. Fredrickson , “Asilomar and Recombinant DNA: The End of the Beginning,” in K.E. Hanna ed., Biomedical Politics (The National Academies Press, 1991): 258–298, at 258–260. 25121217

[147] “Study Sections” National Institute of Health, Center for Scientific Review, April 4, 2022, https://public.csr.nih.gov/StudySections (last visited June 1, 2024).

[148] See Fredrickson, *supra* note 146.

[149] 43 Fed. Reg. at 56192.

[150] *Id*. at 56174.

[151] *Id.* at 56192,

[152] *Id.*

[153] S.L. Schwarcz , “Private Ordering,” Northwestern University Law Review 97, no. 1 (2002): 319–349, at 324.

[154] L. Windsor et al., “Protection of Participants in Community-Engaged Research by Institutional Review Boards: A Call for Action,” American Journal of Public Health 114, no. 55, supp. 5 (2024): s360–s365, at s360. 38547467 10.2105/AJPH.2024.307592 PMC11111368

[155] *Id.* at s363.

[156] See Carter et al., *supra* note 98, at 408.

[157] See Evans and Bihorac, *supra* note 97 at 5.

[158] See “CrowdLaw Catalog,” GovLab, https://catalog.crowd.law (last visited June 1, 2024) (listing 100 examples where digital democracy tools were used to elicit public involvement in contested policy decisions); J. Simon, et al., *Digital Democracy: The Tools Transforming Political Engagement* (NESTA, 2017) (surveying digital democracy tools and providing examples of their use to elicit large-scale public engagement); see also C. Horton, “The Simple but Ingenious System Taiwan Uses to Crowd-Source its Laws” (August 21, 2018), https://www.technologyreview.com/2018/08/21/240284/the-simple-but-ingenious-system-taiwan-uses-to-crowdsource-its-laws/ (last visited June 1, 2024) (discussing Taiwan, one of the most frequently cited digital democracy efforts).

[159] See “Input Crowd, Output Meaning,” Polis, https://pol.is/home (last visited June 1, 2024).

[160] See J. Simon et al., *supra* note 158.

[161] See Evans & Bihorac, *supra* note 97, at 5.

[162] See Muller et al., *supra* note 99, at 7.

